# The Loss of Species: Mangrove Extinction Risk and Geographic Areas of Global Concern

**DOI:** 10.1371/journal.pone.0010095

**Published:** 2010-04-08

**Authors:** Beth A. Polidoro, Kent E. Carpenter, Lorna Collins, Norman C. Duke, Aaron M. Ellison, Joanna C. Ellison, Elizabeth J. Farnsworth, Edwino S. Fernando, Kandasamy Kathiresan, Nico E. Koedam, Suzanne R. Livingstone, Toyohiko Miyagi, Gregg E. Moore, Vien Ngoc Nam, Jin Eong Ong, Jurgenne H. Primavera, Severino G. Salmo, Jonnell C. Sanciangco, Sukristijono Sukardjo, Yamin Wang, Jean Wan Hong Yong

**Affiliations:** 1 IUCN Species Programme/SSC/Conservation International Global Marine Species Assessment, Biological Sciences, Old Dominion University, Norfolk, Virginia, United States of America; 2 Center for Global Trends, The Nature Conservancy, Arlington, Virginia, United States of America; 3 School of Biological Sciences, University of Plymouth, Plymouth, United Kingdom; 4 Centre for Marine Studies, University of Queensland, St. Lucia, Queensland, Australia; 5 Harvard Forest, Harvard University, Petersham, Massachusetts, United States of America; 6 School of Geography and Environmental Studies, University of Tasmania, Launceston, Tasmania, Australia; 7 New England Wild Flower Society, Framingham, Massachusetts, United States of America; 8 Department of Forest Biological Sciences, University of the Philippines Los Baños College, Laguna, Philippines; 9 Centre of Advanced Study in Marine Biology, Annamalai University, Parangipettai, India; 10 Faculty of Sciences and Bioengineering Sciences, Vrije Universiteit Brussel, Brussels, Belgium; 11 Faculty of Liberal Art, Department of Regional Management, Tohoku-Gakuin University, Sendai, Japan; 12 Department of Biological Sciences and Jackson Estuarine Laboratory, University of New Hampshire, Durham, New Hampshire, United States of America; 13 Faculty of Forestry, Nong Lam University, Ho Chi Minh City, Vietnam; 14 Centre for Marine and Coastal Studies, Universiti Sains Malaysia, Penang, Malaysia; 15 Aquaculture Department, Southeast Asian Fisheries Development Center, Tigbauan, Iloilo, Philippines; 16 College of Agriculture, Central Luzon State University, Science City of Munoz, Nueva Ecija, Philippines; 17 Center for Oceanological Research and Development, Indonesian Institute of Sciences, Jakarta, Indonesia; 18 College of Ocean, Shandong University, Weihai, China; 19 Natural Sciences and Science Education Academic Group, National Institute of Education, Nanyang Technological University, Singapore, Singapore; Stanford University, United States of America

## Abstract

Mangrove species are uniquely adapted to tropical and subtropical coasts, and although relatively low in number of species, mangrove forests provide at least US $1.6 billion each year in ecosystem services and support coastal livelihoods worldwide. Globally, mangrove areas are declining rapidly as they are cleared for coastal development and aquaculture and logged for timber and fuel production. Little is known about the effects of mangrove area loss on individual mangrove species and local or regional populations. To address this gap, species-specific information on global distribution, population status, life history traits, and major threats were compiled for each of the 70 known species of mangroves. Each species' probability of extinction was assessed under the Categories and Criteria of the IUCN Red List of Threatened Species. Eleven of the 70 mangrove species (16%) are at elevated threat of extinction. Particular areas of geographical concern include the Atlantic and Pacific coasts of Central America, where as many as 40% of mangroves species present are threatened with extinction. Across the globe, mangrove species found primarily in the high intertidal and upstream estuarine zones, which often have specific freshwater requirements and patchy distributions, are the most threatened because they are often the first cleared for development of aquaculture and agriculture. The loss of mangrove species will have devastating economic and environmental consequences for coastal communities, especially in those areas with low mangrove diversity and high mangrove area or species loss. Several species at high risk of extinction may disappear well before the next decade if existing protective measures are not enforced.

## Introduction

The importance of mangroves for humans and a variety of coastal organisms has been well documented [1]–[7]. Mangrove forests are comprised of unique plant species that form the critical interface between terrestrial, estuarine, and near-shore marine ecosystems in tropical and subtropical regions. They protect inland human communities from damage caused by coastal erosion and storms [8]–[11], provide critical habitat for a variety of terrestrial, estuarine and marine species [5], [12]–[14], and serve as both a source and sink for nutrients and sediments for other inshore marine habitats including seagrass beds and coral reefs [2], [15]. Mangrove species that form dense and often monospecific stands are considered “foundation species” that control population and ecosystem dynamics, including fluxes of energy and nutrients, hydrology, food webs, and biodiversity [16]. Mangroves have been widely reviewed [17] as supporting numerous ecosystem services including flood protection, nutrient and organic matter processing, sediment control, and fisheries. Mangrove forests are the economic foundations of many tropical coastal regions [18] providing at least US$1.6 billion per year in “ecosystem services” worldwide [7]. It is estimated that almost 80% of global fish catches are directly or indirectly dependant on mangroves [1], [19]. Mangroves sequester up to 25.5 million tonnes of carbon per year [20], and provide more than 10% of essential organic carbon to the global oceans [21]. Although the economic value of mangroves can be difficult to quantify, the relatively small number of mangrove species worldwide collectively provide a wealth of services and goods while occupying only 0.12% of the world's total land area [22].

With almost half (44%) of the world's population living within 150 km of a coastline [23], heavily populated coastal zones have spurred the widespread clearing of mangroves for coastal development, aquaculture, or resource use. At least 40% of the animal species that are restricted to mangrove habitat and have previously been assessed under IUCN Categories and Criteria are at elevated risk of extinction due to extensive habitat loss [12]. It is estimated that 26% of mangrove forests worldwide are degraded due to over-exploitation for fuelwood and timber production [24]. Similarly, clearing of mangroves for shrimp culture contributes ∼38% of global mangrove loss, with other aquaculture accounting for another 14% [1]. In India alone, over 40% of mangrove area on the western coast has been converted to agriculture and urban development [25]. Globally, between 20% and 35% of mangrove area has been lost since approximately 1980 [24], [26], [27], and mangrove areas are disappearing at the rate of approximately 1% per year [26], [27], with other estimates as high as 2–8% per year [28]. These rates may be as high as or higher than rates of losses of upland tropical wet forests [24], and current exploitation rates are expected to continue unless mangrove forests are protected as a valuable resource [29].

Given their accelerating rate of loss, mangrove forests may at least functionally disappear in as little as 100 years [2]. The loss of individual mangrove species is also of great concern, especially as even pristine mangrove areas are species-poor compared with other tropical plant ecosystems [29]. However, there is very little known about the effects of either widespread or localized mangrove area loss on individual mangrove species or populations. Additionally, the identification and implementation of conservation priorities for mangroves has largely been conducted in the absence of comprehensive species-specific information, as species-specific data have not been collated or synthesized. Species information including the presence of threatened species is important for refining conservation priorities, such as the designation of critical habitat, no-take zones, or marine protected areas, or to inform policies that regulate resource extraction or coastal development. For the first time, systematic species-specific data have been collated and used to determine the probability of extinction for all 70 known species of mangroves under the Categories and Criteria of the International Union for the Conservation of Nature (IUCN) Red List of Threatened Species.

## Methods

The IUCN Red List Categories and Criteria were applied to 70 species of mangroves, representing 17 families. Hybrids were not assessed as the IUCN Red List Guidelines generally exclude all plant hybrids for assessment unless they are apomicts. Species nomenclature primarily followed Tomlinson [30], and family nomenclature primarily followed Stevens [31], with the exception of Pteridaceae.

The definition of a mangrove species is based on a number of anatomical and physiological adaptations to saline, hypoxic soils. These include viviparous or cryptoviviparous seeds adapted to hydrochory; pneumatophores or aerial roots that allow oxygenation of roots in hypoxic soils; and salt exclusion or salt excretion to cope with high salt concentrations in the peat and pore water in which mangroves grow. Those species that are exclusively restricted to tropical intertidal habitats have been defined as “true mangrove” species, while those not exclusive to this habitat have been termed “mangrove associates” [32]. Tomlinson [30] further subdivided these categories into major mangrove components (true, strict, or specialized mangrove species), minor components (non-specialized mangrove species), and mangrove associates (non-exclusive species that are generally never immersed by high tides). Duke [33] more specifically defined a true mangrove as a tree, shrub, palm, or ground fern generally exceeding 0.5 m in height and which normally grows above mean sea level in the intertidal zone of tropical coastal or estuarine environments. For the IUCN Red List assessments, we defined a mangrove species based on Tomlinson's list of major and minor mangroves, supplemented by a few additional species supported by the expanded definition provided by Duke [33]. It is recognized that the definition used in this study may not strictly apply to all geographic areas. For example, the fern genus *Acrostichum*, which is included in this study, is considered a mangrove associate in some parts of the world [34].

Data collection and assessments for mangrove species probability of extinction were conducted during two IUCN Red List Assessment workshops: one in 2007 in Dominica and the second in 2008 in the Philippines. These two mangrove species assessment workshops brought together 25 of the world's leading mangrove experts to share and synthesize species-specific data, and to collectively apply the IUCN Red List Categories and Criteria [35]. The IUCN Red List Categories and Criteria are the most widely accepted system for classifying extinction risk at the species level [36]–[39]. During the Red List assessment workshops, species were evaluated one at a time by the group of experts present, with outside consultation and follow-up conducted when additional information was needed but not available at the workshop. Information on taxonomy, distribution, population trends, ecology, life history, past and existing threats, and conservation actions for each species was recorded, quantified and reviewed for accuracy. Quantitative species information was then used to determine if a species met the threshold for a threatened category under at least one IUCN Red List Criterion. This IUCN Red List process consolidates the most current and highest quality data available, and ensures peer-reviewed scientific consensus on the probability of extinction for each species [40]–[42]. All species data and results of Red List assessments are freely and publicly available on the IUCN Red List of Threatened Species [43].

The IUCN Red List Categories are comprised of eight different levels of extinction risk: Extinct (EX), Extinct in the Wild (EW), Critically Endangered (CR), Endangered (EN), Vulnerable (VU), Near Threatened (NT), Least Concern (LC) and Data Deficient (DD). A species qualifies for one of the three threatened categories (CR, EN, or VU) by meeting the threshold for that category in one of the five different available criteria (A–E). These different criteria form the real strength of the IUCN Red List as they are based on extinction risk theory [44] and provide a standardized methodology that is applied consistently to any species from any taxonomic group [40]–[42], [45].

Criterion A measures extinction risk based on exceeding a threshold of population decline (30% for Vulnerable, 50% for Endangered, and 80% for Critically Endangered) over a timeframe of three generation lengths, a measure of reproductive turnover rate, in the recent past. To determine if a species could be assessed under Criterion A, percent decline was calculated for each species based on country-level estimates of mangrove area loss between 1980 and 2005 [27] within the species range. Mangrove species generation length, defined as the median age of a reproducing individual based on the estimated age at earliest reproduction and the estimated age at oldest reproduction [35], was conservatively estimated to range between 10 and 40 years based on recent aging techniques developed for *Rhizophora*, *Avicennia* and *Sonneratia* spp. [46]–[48]. As few or no data are available to estimate generation length for all of the mangrove species in this study, the lowest value (10 years) was used uniformly, based on an assumption that mangrove species reproduce throughout their lifetime and can live to an age of at least 15 to 20 years. The two Acrostichum species may be the exception, as stands can live to at least 15 to 20 years, but not likely individual plants. However, this has no bearing on the results for these two species, as declines over the minimum time period required under Criterion A (10 years) do not meet the threshold for a threatened category.

Data based on mangrove area declines from 1980 to 2005 [27] fall within the maximum time frame of three generation lengths estimated for mangroves (30 years) allowed under Criterion A. However, the relationship between mangrove area loss and species range and population reduction is rarely linear, as mangrove area loss can occur in areas of lower or higher population density, and therefore can represent a slower or faster decline of the actual population size [49]. In some cases, area loss can be preceded by impoverishment, due to general decrease in the quality of the forest, or due to specific harvesting of highly prized species like *Rhizophora* spp, which can lead to an underestimation of the rate of disappearance of certain species. Similarly, as the margin-to-area ratio in mangroves is high, not only can deterioration occur rapidly, but changes can occur before areal decreases are detectable [50], including species declines or changes in community composition. By contrast, some species are pioneering or are able to re-colonize rapidly after disturbance. For this reason, expert knowledge and data on the life history traits of each mangrove species, including growth rate and propagation rate, generalized abundance, and where possible, data on pre-1980 declines or continued severity of threats within a restricted geographic distribution were used in combination with mangrove area decline within a species range to estimate and quantify a species' global population decline under Criterion A.

Criterion B measures extinction risk based on a small geographic range size (extent of occurrence <20,000 km^2^ or area of occupancy <2,000 km^2^ to meet the lowest threshold for Vulnerable) combined with continued decline and habitat fragmentation. The majority of species assessed under Criterion B for example, had an area of occupancy estimated to be less than 2,000 km^2^ due to very specific habitat requirements, such as freshwater-dominated river margins or patchy distributions. However, as many mangrove areas are often patchily distributed over considerable distances, estimations of area of occupancy or extent of occurrence for mangrove species were conservative. Criterion C is applied to species with small population sizes estimated to be less than 10,000 mature individuals, with continued decline. Although not used to assess mangrove species in this study, Criterion D is applied to species with less than 1,000 mature individuals or those with an area of occupancy of less than 20 km^2^, and Criterion E is applied to species with extensive population information that allows for population declines to be appropriately modeled over time. A category of Near Threatened is assigned to species that come close to but do not fully meet the all the thresholds or conditions required for a threatened category under Criterion A, B, C, D or E.

## Results and Discussion

Of the 70 species of true mangrove species, 11 (16%) qualified for one of the three Red List categories of threat: Critically Endangered, Endangered, or Vulnerable (Table 1). *Heritiera* is the genus with the most threatened mangrove species with 2 of the 3 species (66%) in threatened categories. Seven species (10%) only partially met the thresholds for a threatened category and were therefore listed as Near Threatened. Four species (6%) were listed as Data Deficient primarily due to critical gaps in knowledge of the extent of the species distribution *e.g., Acanthus xiamenensis* from the extensively developed and heavily polluted estuaries of the Jiulong River in Fujian Province, China [53]. A listing of Data Deficient does not preclude future listing of the species when more data are gathered. Forty-eight species (68%) were assessed as Least Concern. Even though mangrove area continues to be lost where the majority of these “Least Concern” species are found [27], the global population decline over the past 30 years for each of these species was estimated to be below the threshold required for assignment to a threatened category. Some of the “Least Concern” species also are considered to be common, fast-growing, early-successional species.

**Table 1:** Mangrove species, Red List Categories and Criteria, and summary of supplemental data (CR = Critically Endangered, EN = Endangered, VU = Vulnerable, NT = Near Threatened, LC = Least Concern, DD = Data Deficient).

		Red List	Criterion	Global	Estuarine			Intertidal			Supporting Information:
Family	Species	Category	Applied*	% Loss	Position			Position			Generalized Abundance and Life History
ACANTHACEAE	*Acanthus ebracteatus*	LC		22		I			M	H	common
ACANTHACEAE	*Acanthus ilicifolius*	LC		20		I	U		M	H	common
ACANTHACEAE	*Acanthus volubilis*	LC		24			U			H	uncommon
ACANTHACEAE	*Acanthus xiamenensis*	DD		34	?	?	?	?	?	?	unknown, distribution not well-known
ACANTHACEAE	*Avicennia alba*	LC		24	D			L	M		locally common, fast-growing, colonizing
ACANTHACEAE	*Avicennia bicolor*	VU	A	31	D					H	uncommon
ACANTHACEAE	*Avicennia germinans*	LC		17	D	I			M	H	locally common
ACANTHACEAE	*Avicennia integra*	VU	B	<5		I		L			<5000 individuals, extent of occurrence<20,000 km^2^
ACANTHACEAE	*Avicennia marina*	LC		21	D	I		L	M	H	common, fast growing, colonizing
ACANTHACEAE	*Avicennia officinalis*	LC		24		I			M		common, fast growing, colonizing
ACANTHACEAE	*Avicennia rumphiana*	VU	A	30	D					H	uncommon to rare, patchy distribution
ACANTHACEAE	*Avicennia schaueriana*	LC		6	D				M	H	locally common
ARECACEAE	*Nypa fruticans*	LC		20			U	L	M	H	common
ARECACEAE	*Phoenix paludosa*	NT	B	14			U			H	uncommon, area of occupancy<2000 km^2^
BIGNONIACEAE	*Dolichandrone spathacea*	LC		23			U		M		uncommon, fast-growing
BIGNONIACEAE	*Tabebuia palustris*	VU	A	33			U	L	M		rare, narrow habitat range, cryptic
COMBRETACEAE	*Conocarpus erectus*	LC		17	D					H	common
COMBRETACEAE	*Laguncularia racemosa*	LC		17	D	I			M	H	locally common, pioneering
COMBRETACEAE	*Lumnitzera littorea*	LC		22		I			M		common
COMBRETACEAE	*Lumnitzera racemosa*	LC		19	D				M	H	common, colonizing, fast-growing
EBENACEAE	*Diospyros littorea*	LC		24		I	U		M	H	uncommon
EUPHORBIACEAE	*Excoecaria agallocha*	LC		21	D	I	U		M	H	common, tolerant of disturbed areas
EUPHORBIACEAE	*Excoecaria indica*	DD		24	D	I		L	M		unknown, distribution not well-known
FABACEAE	*Cynometra iripa*	LC		21		I	U			H	locally common, slow-growing
FABACEAE	*Mora oleifera*	VU	C	26			U			H	<10000 individuals
LYTHRACEAE	*Pemphis acidula*	LC		21	D					H	common, locally threatened by bonsai trade
LYTHRACEAE	*Sonneratia alba*	LC		20	D			L			common, fast-growing, pioneering, low seed viability
LYTHRACEAE	*Sonneratia apetala*	LC		7			U	L	M		common, fast-growing, pioneering, low seed viability
LYTHRACEAE	*Sonneratia caseolaris*	LC		20			U	L			common, fast-growing, pioneering, low seed viability
LYTHRACEAE	*Sonneratia griffithii*	CR	A	80	D			L			rare, locally extinct, low seed viability
LYTHRACEAE	*Sonneratia lanceolata*	LC		24			U	L			locally common, fast-growing, pioneering, low seed viability
LYTHRACEAE	*Sonneratia ovata*	NT	A	28	D					H	locally common, low seed viability, severe loss at range extremities
MALVACEAE	*Brownlowia argentata*	DD		26			U			H	unknown, distribution not well-known
MALVACEAE	*Brownlowia tersa*	NT	A	26			U			H	locally common, severe loss at range extremities
MALVACEAE	*Camptostemon philippinense*	EN	C	30		I		L			<1200 individuals
MALVACEAE	*Camptostemon schultzii*	LC		24	D	I		L	M		locally common, uncommon,
MALVACEAE	*Heritiera fomes*	EN	A	50–80			U			H	common to uncommon, slow-growing
MALVACEAE	*Heritiera globosa*	EN	B	29			U			H	rare, extent of occurrence <5,000 km^2^
MALVACEAE	*Heritiera littoralis*	LC		20		I				H	common
MELIACEAE	*Aglaia cucullata*	DD		23			U		M		unknown, distribution not well-known
MELIACEAE	*Xylocarpus granatum*	LC		21		I			M	H	common, slow-growing
MELIACEAE	*Xylocarpus moluccensis*	LC		21			U			H	common, slow-growing
MYRSINACEAE	*Aegiceras corniculatum*	LC		21		I	U	L			common
MYRSINACEAE	*Aegiceras floridum*	NT	A	29	D			L			uncommon, narrow habitat tolerance
MYRTACEAE	*Osbornia octodonta*	LC		23	D				M	H	uncommon, slow-growing, hardy
PLUMBAGINACEAE	*Aegialitis annulata*	LC		24	D				M	H	common
PLUMBAGINACEAE	*Aegialitis rotundifolia*	NT	B	24	D				M	H	rare, area of occupancy<2000 km^2^
PTERIDACEAE	*Acrostichum aureum*	LC		19		I				H	common, fast-growing, hardy, colonizing
PTERIDACEAE	*Acrostichum danaeifolium*	LC		17		I				H	unknown, distribution not well-known
PTERIDACEAE	*Acrostichum speciosum*	LC		21		I	U			H	common, fast-growing, hardy, colonizing
RHIZOPHORACEAE	*Bruguiera cylindrica*	LC		24	D	I			M	H	common, high regeneration potential, slow growth rate
RHIZOPHORACEAE	*Bruguiera exaristata*	LC		23		I	U		M	H	common
RHIZOPHORACEAE	*Bruguiera gymnorhiza*	LC		20	D	I			M	H	common, slow-growing, low regeneration
RHIZOPHORACEAE	*Bruguiera hainesii*	CR	C	27		I				H	∼250 individuals, slow-growing, low propagation and germination
RHIZOPHORACEAE	*Bruguiera parviflora*	LC		21	D	I			M		common, slow-growing
RHIZOPHORACEAE	*Bruguiera sexangula*	LC		21		I	U		M	H	uncommon, slow-growing
RHIZOPHORACEAE	*Ceriops australis*	LC		24	D	I				H	common, slow-growing, hardy
RHIZOPHORACEAE	*Ceriops decandra*	NT	B	12		I			M	H	rare, slow-growing, area of occupancy <4500 km^2^
RHIZOPHORACEAE	*Ceriops tagal*	LC		18	D	I			M	H	common, slow-growing, hardy
RHIZOPHORACEAE	*Ceriops zippeliana* [52]	LC		23		I			M	H	common, slow-growing
RHIZOPHORACEAE	*Kandelia candel*	LC		23	D			L			locally common, lower regeneration, hardy
RHIZOPHORACEAE	*Kandelia obovata*	LC		29	D			L			common, hardy, easily propagated, range increasing in Japan
RHIZOPHORACEAE	*Rhizophora apiculata*	LC		20		I			M		very common, hardy, fast-growing
RHIZOPHORACEAE	*Rhizophora mangle*	LC		17	D	I		L	M		common, hardy, fast-growing
RHIZOPHORACEAE	*Rhizophora mucronata*	LC		20		I	U	L	M		common, hardy, fast-growing
RHIZOPHORACEAE	*Rhizophora racemosa*	LC		15	D	I			M		locally common, can form large stands with patch distribution
RHIZOPHORACEAE	*Rhizophora samoensis*	NT	A	29	D	I		L	M		common to rare, locally threatened by intensive harvesting
RHIZOPHORACEAE	*Rhizophora stylosa*	LC		20	D	I		L	M		common, hardy, fast-growing
RUBIACEAE	*Scyphiphora hydrophylacea*	LC		20		I				H	uncommon
TETRAMERISTACEAE	*Pelliciera rhizophorae*	VU	B	27		I	U		M	H	relict and rare, area of occupancy<2000 km^2^

Estuarine (D = downstream, I = intermediate, U = upstream) and intertidal (L = low, M = medium, H = high) positions are modified from [51].

*see main text for criterion definitions.

Of special concern are the two species that are listed as Critically Endangered, the highest probability of extinction measured by the IUCN Red List. The rare *Sonneratia griffithii* is distributed in parts of India and southeast Asia, where a combined 80% loss of all mangrove area has occurred within its patchy range over the past 60 years, with significant losses in Malaysia [54], primarily due to the clearing of mangrove areas for rice farming, shrimp aquaculture, and coastal development [55]. This species is already reported to be locally extinct in a number of areas within its range, and less than 500 mature individuals are known from India. *Bruguiera hainesii* is an even rarer species and is only known from a few fragmented locations in Indonesia, Malaysia, Thailand, Myanmar [56], Singapore and Papua New Guinea. It has very low rates of propagation and low rates of germination. It is estimated that there are less than 250 mature individuals remaining. For these species, urgent protection is needed for remaining individuals as well as research to determine minimum viable population size.

All but two species that were listed in threatened categories (Critically Endangered, Endangered or Vulnerable) or as Near Threatened are rare or uncommon, and/or have small population sizes. The two Near Threatened species, *Sonneratia ovata* and *Brownlowia tersa*, are common throughout their relatively wide range, but have experienced severe loss at their range margins. It is well established that rare species (species with very small population or range size, low abundance, and/or associated specialist pattern of resource use [57]), have a higher intrinsic risk of extinction [58], [59]. As the IUCN Red List Criteria are based on extinction risk, the quantitative thresholds for each threatened category are designed to capture these rare or uncommon species with small ranges and/or low population sizes.

Being uncommon, however, is not always a precursor to being threatened. At least five of the species listed as Least Concern are considered uncommon. General abundance was only one factor that was used to interpret global population status for a species in combination with overall area loss in a species range and other life history traits. For example, a species with a low risk of extinction can be uncommon, but can grow over a very large range with little or low mangrove area loss. Alternately, it can be uncommon and exist in a smaller range or degraded area, but be fast-growing, hardy and easily propagated, (e.g., more of a habitat generalist). However, mangrove species abundance generally is not the same across the entire range of a given species, as mangrove species tend to be less common at their range margins and can be locally abundant where salinity or other environmental factors are closer to each species' optimum [51].

Many mangrove forests exhibit distinct zones of species that are controlled by the elevation of the substrate relative to mean sea level and the associated variation in frequency of elevation, salinity and wave action [51]. Such zonation is not always apparent [60] and may be disrupted by anthropogenic disturbance. Seven of 16 (44%) mangrove species found primarily in the upstream estuarine or high intertidal region are in threatened or Near Threatened categories. These species have very specific freshwater-dominated habitat requirements, are often patchily distributed, and are often occupy areas that are cleared first for the construction of shrimp or fish ponds, or for agriculture. For example, populations of the Endangered *Heritiera fomes* and *Heritiera globosa* in Southeast Asia have been severely reduced due to coastal development, the creation of ponds, reduction of freshwater from the creation of dams, and expansion of palm and timber plantations. Three of 10 (30%) of mangrove species found primarily in the downstream estuarine and low intertidal region are in threatened or Near Threatened categories. Many of these fringe species, such as the Near Threatened *Aegiceras floridum*, have high salinity requirements, and are only found along beaches and in rocky or sandy substrate [61]. In these mangrove areas, populations are experiencing severe declines due to coastal development and the conversion of tidal wetlands to fish ponds or other land uses.

The primary threats to all mangrove species are habitat destruction and removal of mangrove areas for conversion to aquaculture, agriculture, urban and coastal development, and overexploitation. Of these, clear-felling, aquaculture and over-exploitation of fisheries in mangroves are expected to be the greatest threats to mangrove species over the next 10–15 years [29]. Climate change is also considered a threat to all mangrove species, particularly at the edges of a species range where sea temperature and other environmental changes may be greatest. With a rise in sea level, the habitat requirements of each species will be disrupted, and species zones will suffer mortality in their present tidal zones and attempt to re-establish at higher elevations in areas that were previously landward zones [62]. Mangrove species with a habitat on the landward margin are particularly vulnerable to sea-level rise if, owing to coastal development, their movement inland is blocked. Species that occur at the landward edge, or upstream in tidal estuaries include *Brownlowia tersa*, *Bruguiera sexangula*, *Nypa fruticans*, *Phoenix paludosa*, *Lumnitzera racemosa*, *Lumnitzera littorea*, *Sonneratia caseolaris*, *Sonneratia lanceolata*, and *Xylocarpus granatum*. Species that are easily dispersed, and grow or reproduce rapidly, such as *Rhizophora* spp may cope better than those which are slower growing and slower to reproduce such as *Bruguiera* spp, *Ceriops* spp, or *Xylocarpus* spp.

### Geographic Areas of Concern

**Figure 1:**
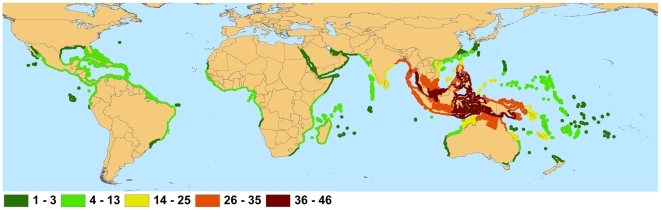
Mangrove Species Richness: Native distributions of mangrove species. Not shown are introduced ranges: *Rhizophora stylosa* in French Polynesia, *Bruguiera sexangula*, *Conocarpus erectus*, and *Rhizophora mangle* in Hawaii, *Sonneratia apelata* in China, and *Nypa fruticans* in Cameroon and Nigeria.

**Figure 2:**
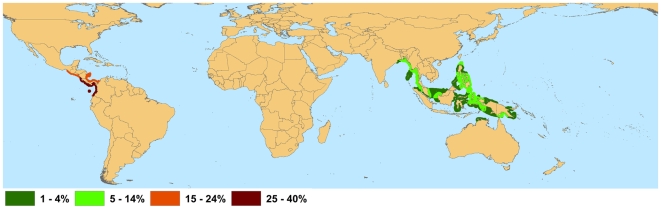
Proportion of Threatened (Critically Endangered, Endangered, and Vulnerable) Mangrove Species.

Range declines for all mangrove species from habitat loss and localized threats are occurring in all tropical coastal regions of the world [27]; however, some regions show greater losses than others. Unlike many other forests, mangrove forests consist of relatively few species, with thirty to forty species in the most diverse sites and only one or a few in many places [51]. Globally, mangrove biodiversity is highest in the Indo-Malay Philippine Archipelago (Figure 1), with between 36 and 46 of the 70 known mangrove species occurring in this region. Although less than 15% of species present in this region are in threatened categories (Figure 2), the Indo-Malay Philippine Archipelago has one of the highest rates of mangrove area loss globally, with an estimated 30% reduction in mangrove area since 1980 [27]. Mangroves in this region are primarily threatened by clearing for the creation of shrimp and fish ponds [63], for example, approximately half of the 279,000 ha of mangroves in the Philippines lost from 1951 to 1988 were developed into fish/shrimp culture ponds [64]. *Camptostemon philippinense*, listed as Endangered, has an estimated 1200 or less individuals remaining due to the extensive removal of mangrove areas for both aquaculture and fuelwood within its range. The Endangered *Heritiera globosa* has the most restricted distribution in this region (extent of occurrence<5,000 km^2^) as it is only known from western Borneo, where its patchily distributed, primarily riverine habitat has been extensively cleared by logging activities and for the creation of timber and oil palm plantations.

Geographic areas with a high numbers of mangrove species at elevated risk of extinction are likely to exhibit loss of ecosystem function, especially in areas of low mangrove diversity. Globally, the highest proportion of threatened mangrove species is found along the Atlantic and Pacific coasts of Central America (Figure 2). Four of the 10 (40%) mangrove species present along the Pacific coasts of Costa Rica, Panama and Colombia are listed in one of the three threatened categories, and a fifth species *Rhizophora samoensis* is listed as Near Threatened. Three of these species, *Avicennia bicolor*, *Mora oleifera* and *Tabebuia palustris* all listed as Vulnerable, are rare or uncommon species only known from the Pacific coast of Central America. Extensive clearing of mangroves for settlement, agriculture and shrimp ponds are the major causes of mangrove decline in Latin America [65], even though there is little compensating economic return from conversion of mangrove areas to agriculture [66].

After the Indo-Malay Philippine Archipelago, the Caribbean region has the second highest mangrove area loss relative to other global regions, with approximately 24% of mangrove area lost over the past quarter-century [27]. Several surveys of Caribbean mangroves report significant regional declines due to a myriad of threats including coastal development, upland runoff of pollutants, sewage, and sediments, petroleum pollution, storms and hurricanes, solid waste, small-scale extraction for fuelwood and minor clearcutting, conversion to aquaculture, conversion to landfills, conversion for terrestrial agriculture, tourism (involving construction of boardwalks and moorings, as well as boat wakes), and prospecting for pharmaceuticals [67], [68]. However, with the exception of the Central American endemic *Pelliciera rhizophorae* listed as Vulnerable, the 8 other mangrove species present in the Caribbean region did not qualify for a threatened category because they are relatively widespread and found in other regions such as West Africa or Brazil. After Indonesia, Australia, and Mexico, Brazil has the fourth largest area of mangroves [27], [69], and although some areas are affected by aquaculture, human settlement and water pollution [70], [71], there has been very little estimated mangrove area loss in Brazil since 1980 [27].

Mangrove diversity is naturally low at the northern and southern extremities of mangrove global range, such as southern Brazil, the Arabian Peninsula, and the northern and southern Atlantic coasts of Africa, as well as on islands in the South Pacific [72] and the Eastern Tropical Pacific. Although the majority of species present at these extremes of mangrove global distribution have very widespread global ranges, and have not been listed in threatened categories, populations are more at risk from area declines at these extremes of their distribution where mangrove diversity is lowest [51].

### The Cost of Mangrove Species Loss

The loss of individual mangroves species and associated ecosystem services has direct economic consequences for human livelihoods, especially in regions with low mangrove species diversity and low ecosystem resilience to species loss. In the Gulf of California, for example, where there are only 4 mangrove species present (*Avicennia germinans*, *Rhizophora samoensis*, *Laguncularia racemosa*, *Conocarpus erectus*), it is estimated that one linear kilometer of the species *R. samoensis*, listed as Near Threatened, provides up to 1 ha of essential marine habitat and provides a median annual value of US$37,000 in the fish and blue crab fisheries [73].

Nutrients and carbon from mangrove forests provide essential support to other near shore marine ecosystems such as coral reefs and seagrass areas, and enrich coastal food webs and fishery production [1], [28]. *Avicennia* species are dominant in inland or basin mangrove forests in many parts of the world. However, 3 of 8 (38%) species in this genus are in threatened or Near Threatened categories. Loss of these species and the mangrove forests they dominate will have far reaching consequences for water quality and other near shore ecosystems in coastal communities around the globe. For example, water purification services provided by these mangrove species in the Muthurajawela Marsh, Sri Lanka are valued at more than $US 1.8 million per year [74].

Riverine or freshwater-preferring species, such as the Endangered *Heritiera fomes* and *Heritiera globosa*, buffer coastal rivers and freshwater communities from sedimentation, erosion and excess nutrients. *Heritiera globosa* is a very rare species confined to western Borneo, while *Heritiera fomes* is more widespread in south Asia, but has experienced significant declines in many parts of its range. Localized or regional loss of these coastal or fringe mangrove species reduces protection for coastal areas from storms, erosion, tidal waves, and floods [6], [9], with the level of protection also dependent on the quality of remaining habitat [8]. Two of 4 (50%) fringe mangrove species present in Southeastern Asia (*Sonneratia griffithii*, *Aegiceras floridum*) are listed in threatened or Near Threatened categories. In other areas, such as Brazil, the central Pacific islands, or West Africa, fringe mangrove forests are often comprised of only one or two species. Even though these species are globally listed as Least Concern, local and regional loss of mangroves in these areas will have devastating impacts for coastal communities. The loss of species may indeed be of greatest economic concern in rural, high-poverty areas where subsistence communities rely on mangrove areas for fishing and for direct harvesting of mangroves for fuel, construction or other economic products [17], [75]–[77].

Finally it is important to note that the amount of mangrove area in some countries is increasing due to reforestation and restoration efforts [27], [29]. Although regeneration of degraded mangrove areas is thought to be a viable option in some areas [17], [78], successful regeneration is generally only achieved by the planting of monocultures of fast-growing species, such as *Rhizophora or Avicenna* species. Many rare and slow growing species are not replaced [29], and many species cannot be easily replanted with success. In sum, mangrove areas may be able to be rehabilitated in some regions, but species and ecosystems cannot be effectively restored.

### Conclusions and Recommendations

There are currently eleven international treaties and instruments that afford some protection, at least on paper, to mangroves in general, some of which have been in force for over 50 years. These treaties and instruments include the RAMSAR Convention, the Convention on the Prevention of Marine Pollution, CITES, the International Tropical Timber Agreement, the Convention for the Protection and Development of the Marine Environment of the Wider Caribbean Region and the Convention on Biological Diversity [70], [79]. However, these treaties and instruments do not necessarily confer legal protection to mangrove ecosystems, and none of them address conservation, preservation, or management of particular mangrove species. Similarly, the current trend of global decline of mangrove area [27] indicates that exploitation continues unabated despite the presence of these laws and treaties.

With some exceptions, mangrove areas and species of concern are generally not adequately represented within protected areas. In addition to legislative actions, initiatives are needed on the part of governments, NGOs, and private individuals to acquire and protect privately-owned parcels of coastal land, especially those that contain viable populations of threatened mangrove species. National legislation and management plans are in place in some countries, but enforcement and further planning are required to protect individual species that may be locally uncommon or threatened, as well as to protect the entire mangrove areas and important ecosystem functions.

IUCN Red List assessments for species can be regularly updated, depending on the availability of better or new data, and any subsequent changes in a species Red List Category can be an important indicator of the success or failure of conservation actions. As the impacts of mangrove area loss on mangrove species can be variable, estimation of species composition, individual species decline, or population size in a given area can be better refined by available remote sensing techniques [80]–[85]. Similarly, demographic modeling [86] is needed to establish a minimum viable population size for mangrove species, especially for those that are highly threatened. As ecosystem values can be overestimated or underestimated, additional studies and cost/benefit analyses are needed to determine the economic and ecological impacts of harvesting, habitat loss, and habitat deterioration on populations of individual mangrove species.

At least two mangrove species are at high risk of extinction and may disappear within the next decade if protective measures are not enforced. Although not formally assessed by IUCN Red List Categories and Criteria, hybrid species face the same threats and provide ecosystem services equivalent to true mangrove species. Their conservation should not be overlooked, especially as they are important for speciation and can be significant drivers of diversification over time. We maintain that the loss of individual species will not only contribute to the rapid loss of biodiversity and ecosystem function, but will also negatively impact human livelihoods and ecosystem function, especially in areas with low species diversity and/or high area loss.
